# What is the real physiological NO concentration *in vivo*?

**DOI:** 10.1016/j.niox.2009.07.002

**Published:** 2009-09-15

**Authors:** Catherine N. Hall, John Garthwaite

**Affiliations:** a Department of Neuroscience, Physiology and Pharmacology, University College London, UK; b Wolfson Institute of Biomedical Research, University College London, Gower St., London WC1E 6BT, UK

**Keywords:** Nitric oxide, Guanylyl cyclase, cGMP, Cytochrome *c* oxidase, Mitochondria, Inflammation, Excitotoxicity

## Abstract

Clarity about the nitric oxide (NO) concentrations existing physiologically is essential for developing a quantitative understanding of NO signalling, for performing experiments with NO that emulate reality, and for knowing whether or not NO concentrations become abnormal in disease states. A decade ago, a value of about 1 μM seemed reasonable based on early electrode measurements and a provisional estimate of the potency of NO for its guanylyl cyclase-coupled receptors, which mediate physiological NO signal transduction. Since then, numerous efforts to measure NO concentrations directly using electrodes in cells and tissues have yielded an irreconcilably large spread of values. In compensation, data from several alternative approaches have now converged to provide a more coherent picture. These approaches include the quantitative analysis of NO-activated guanylyl cyclase, computer modelling based on the type, activity and amount of NO synthase enzyme contained in cells, the use of novel biosensors to monitor NO release from single endothelial cells and neurones, and the use of guanylyl cyclase as an endogenous NO biosensor in tissue subjected to a variety of challenges. All these independent lines of evidence suggest the physiological NO concentration range to be 100 pM (or below) up to ∼5 nM, orders of magnitude lower than was once thought.

## Introduction

It is more than 20 years since nitric oxide (NO) emerged as a biological signalling molecule, initially in the cardiovascular, nervous and immune systems [1–4] and then almost everywhere else as well [5]. Were it a conventional type of hormone or transmitter, its principal modes of operation should be well understood by now. For example, over a similar time frame, the amino acid glutamate progressed from being simply a putative neurotransmitter in the central nervous system to being recognized as the major excitatory neurotransmitter therein [6] with complex primary actions in synapses that can now be accurately simulated in sub-micrometer dimensions and on a sub-millisecond time scale [7]. With NO, achieving this degree of clarity still seems a rather distant prospect.

The mechanisms of NO formation by the three NO synthase enzymes (neuronal, endothelial and inducible, or nNOS, eNOS and iNOS) have become quite well established and their functioning in a cell-free environment can be modelled with a good degree of accuracy [8]. Beyond that there lacks a coherent empirical or conceptual framework for how NO operates physiologically. One drawback is that the lack of chemical specialization of the NO molecule renders fairly hopeless any attempt to exploit agonist/antagonist-based pharmacology that has traditionally been so profitable. Moreover, a molecule with the physicochemical properties of NO had not been encountered previously in the field of biological signalling, necessitating the formulation of a somewhat different set of rules for how it works. Most obviously, NO diffuses freely and very rapidly in three-dimensions away from its point of synthesis rather than being spatially constrained in the intracellular or extracellular environment by membranes. How far does it spread in biologically relevant concentrations? What is the profile of NO release over time from the different sources? How is it captured to elicit biological responses? The answers to these key questions remain matters of speculation and debate. Moreover, depending on its concentration and/or the period of exposure, NO may exert multiple effects ranging from activation of its guanylyl cyclase (GC)-coupled receptor proteins, through to inhibition of cellular respiration by binding to cytochrome *c* oxidase in mitochondria, through to participation in various chemical reactions.

Where physiology begins and ends within this spectrum of potential activities is probably the most important question in the field as a whole, and remains the source of much uncertainty and, perhaps also, wishful thinking. Judging from the literature, almost anything from femtomolar [9] up to hundreds of micromolar [10] concentrations can be regarded as physiological. The lack of consensus means that there are few constraints on the amounts of NO that are applied to cells or tissues, making it difficult to discriminate results that have physiological or pathological significance from those that are biologically meaningless. Clarity about this issue is vital for the development of a quantitative understanding of the principles of NO signaling, for designing experiments using exogenous or endogenous sources of NO that emulate reality, and for knowing if and when NO concentrations become abnormal. Here we scrutinise the evidence and arrive at some broad conclusions. Physiological NO concentrations are taken to be those that are not, generally speaking, injurious and which are found during normal tissue functioning, although reference to pathological situations is also included.

## Attempts at direct measurement

**Fig. 1:**
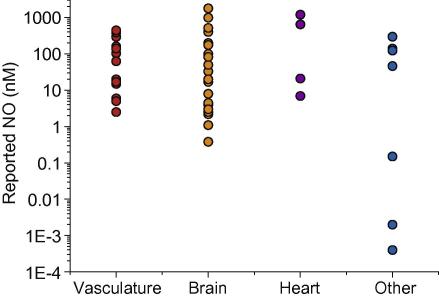
NO concentrations reported for different tissues. Data are from Table 1.

Direct electrochemical detection of NO has been used for many years to try to measure the concentration of NO in cells and tissues, both basally and in response to various stimulators of NO production. Direct determination of NO concentrations would be expected to provide the best solution but the results have been disappointing. One only need look at the huge variability in tissue NO concentrations reported by different groups using different electrodes to appreciate the scale of the problem: the range covers 10^6^ orders of magnitude (Fig. 1 and Table 1). Even for a given tissue the variability is improbably large. In arteries, for example, NO concentrations produced *in vitro* on application of acetylcholine have ranged from 2.5 to 390 nM [11,12]. Similarly, ischaemia in rat brain *in vivo* has been reported to raise NO by 17 nM or 1 μM [13,14].

One reason for such a wide spread of recorded endogenous NO concentrations is likely to be cross-reactivity of some electrodes with other biological species. Specificity of microelectrodes for NO comes, in part, from the redox potential for NO oxidation but as other biological species, for instance nitrite, dopamine, 5-hydroxytryptamine (5-HT) and tyrosine are oxidised at similar potentials, these species are common cross-reactants [15,16]. In aiming to reduce interference, electrodes are coated with membranes such as Nafion, which is impermeable to negatively charged species such as nitrite, and *ο*-phenylenediamine, which reduces interference by cationic substances such as dopamine and 5-HT [17]. The degree of selectivity depends on the number of coatings of these membranes and their integrity [17,18], and day-to-day electrode behaviour can change dramatically [19].

Many authors do not discuss the selectivity of their electrodes, so interpretation of their records is problematic. Additionally, some reported selectivity ratios would be insufficient to discriminate between low levels of NO and physiological levels of potential interferents. For example, Ferreira et al. report a selectivity ratio for NO versus dopamine of 18:1, and versus ascorbate of 750:1 [20]. Brain ascorbate concentrations are estimated to be 500 μM extracellularly, however [21], and this level was found to increase by 250–500 μM on application of NMDA and glutamate [22]. The electrode used by Ferreira et al. would record such a change in ascorbate as 330–660 nM NO. Similarly, neuronal activity increased brain dopamine levels to 500 nM [23], which would be recorded as an apparent NO increase of 28 nM. Ferreira et al. report an increase in NO of 250–350 nM on application of NMDA and glutamate [20], a result that could be accounted for by cross-reactivity with ascorbate and dopamine.

Some studies have attempted to discriminate between NO and interferents, reasoning that only the portion of the signal that is sensitive to NOS inhibition represents NO (e.g. [24]). This approach might also be misleading because the generation of NO and of potential interferents may be interdependent. For example, NO has been shown to regulate dopamine release [25] and to stimulate ascorbate efflux from red blood cells [26]. Testing for interference by physiological concentrations of these contaminants should reduce the possibility of artefact. It may be no coincidence that studies that explicitly report a lack of sensitivity to biologically relevant concentrations of, among others, dopamine, ascorbate and 5-HT, tend to report lower evoked NO concentrations (<10 nM [27–30]). At the lower extreme, that the concentration could be in the femtomolar range [9] seems improbable because no biological effects of NO have been observed in this concentration range and because the electrode design adopted, in the originator’s hands, is 2 million-fold too insensitive (detection limit = 10 nM [31]). A calibration error is suspected.

With such an immense variability of values reported in the literature, it is difficult to come to any firm conclusion. As things stand, the lower reported values are probably closer to the truth, signifying that physiological NO may be <10 nM rather than >100 nM.

## Indirect NO indicators

### Fluorescent probes

Probes that bind NO with a high degree of selectivity and alter their fluorescence as a result, offer the highly desirable prospect of imaging NO concentrations over time and distance in living tissues in a quantitative manner. The most popular have been diamino derivatives of fluorescein (DAF-2) and rhodamine (DAR), which are available as cell permeating acetoxymethyl esters that undergo hydrolysis intracellularly to liberate the active compound. Such compounds are typically marketed as NO detectors and are reputed to have sensitivity to NO in the low nanomolar range [32,33]. Strictly speaking, however, they are not NO detectors because they do not react with NO. Various mechanisms have been proposed to explain the changes in fluorescence in cells and tissues, an attractive one being that of Wardman [34], who suggested that non-specific oxidation of the fluorophore transforms one of the aromatic amino groups into an NH radical; NO binds to the radical, leading to the formation of a triazole ring and a change in fluorescence. This mechanism helps explain many experimental observations of non-specificity/interference and implies that a change in fluorescence, even if prevented by NO synthase inhibition or scavenging, does not necessarily signify a change in NO concentration. Simply a change in the redox state of a cell could alter the degree of oxidation of the fluorophore leading to a change in the amount of the radical species available to react with NO and, hence, a change in fluorescence. Interpretation of the fluorescent signals in terms of NO is therefore problematic and quantifying the signals in terms of NO concentration is not feasible.

A more recent approach has been to make fluorophores complexed with Cu^2+^. Binding of NO causes reduction to Cu^+^, which dissociates, and the formation of NO^+^, which nitrosates a nitrogen atom on the molecule, increasing the fluorescence emission [35–37]. Where studied, the NO detection limit is reported to be 17 nM after 5 min exposure [36]. Fluorescent responses have been detected by imaging in various cells, including endothelial cells and macrophages, but no attempt has yet been made to calibrate the responses so the prevailing NO concentrations cannot be deduced.

### NO biosensors

By incorporating an amplification step, Sato et al. [38,39] have succeeded in designing a biosensor capable of detecting NO in concentrations in the picomolar range. The basis of the sensor is the physiological NO receptor, NO-activated GC. On each of the two GC subunits, Sato et al. positioned a fluorescent cGMP detector that comprised the cGMP binding domain of cGMP-dependent protein kinase together with yellow and cyan fluorescent proteins, yielding a FRET signal on cGMP binding. This approach immediately circumvents problems of cross-reactivity inherent in all the other methods discussed above because the NO receptor is extremely selective for NO. The only other known endogenous ligand for the receptor is carbon monoxide but its affinity and efficacy are very low compared with NO [40,41] and, accordingly, a concentration of 100 μM was needed to give a detectable response [38]; this concentration was a million-fold higher than the detection limit of the sensor for NO (0.1 nM). When cultured vascular endothelial cells were transfected with the sensor, the recorded basal intracellular NO concentration was estimated from calibrations with authentic NO to be 1 nM. Application of mechanical shear stress or 1 nM bradykinin to evoke eNOS activation caused a transiently increased FRET signal (not quantified). The group then generated a stable cell line expressing the NO biosensor and used it to monitor NO released from cells [39]. The cell line, named Piccell, was able to detect astonishingly low NO concentrations, the limit being 20 pM. By positioning a Piccell next to an endothelial cell, or an endothelial cell on top of a Piccell carpet, it was found that stimulation of the endothelial cell by photorelease of ATP transiently produced up to 100 pM NO in the adjacent Piccell, falling to about 30 pM a distance of 20 μm away (estimated from their Fig. 4E). In a similar experiment with a Piccell positioned next to a cultured brain neurone (from the hippocampus), oscillatory NO signals were recorded. From the evidence presented (e.g., sensitivity to the voltage-gated sodium channel blocker, tetrodotoxin), the oscillations probably originated in circuit-based neuronal activity. Similar to endothelial cells, the peak NO concentration reaching the Piccell was about 100 pM.

If correct, these measurements suggest that the physiological NO concentration generated at the surface of an endothelial cell is about 10,000-fold lower than was originally reported by Malinski [31]. The results await confirmation, however, and it is not certain in the case of the neuronal cultures that the NO was derived from the immediately adjacent cell as, judging from the image shown in their Fig. 3C, there appears to be a meshwork of neuronal processes in the vicinity that may be active simultaneously. The recent engineering of cGMP biosensors with improved kinetics [42,43] should provide the means for obtaining complementary information using endogenous GCs as the NO detectors and amplifiers, potentially allowing quantifiable responses to NO in the low nM range and below.

## Deductions from functional evidence

### NO receptor-mediated responses

Knowledge of the kinetics of receptors for signalling molecules reveals much about the messages they have evolved to detect and decode. NO-activated GCs, of which there are two known isoforms in mammals (α1β1 and α2β1), are the only recognized NO receptors and they transduce all the classical actions of NO in relaxing smooth muscle, inhibiting platelet aggregation, and in modulating brain function [44,45]. Despite a long gestation, the functioning of these NO receptors is now becoming understood in quantitative terms, thanks partly to the establishment of simple methods for delivering NO in known, constant concentrations [46] which is a prerequisite for this type of analysis.

**Fig. 2:**
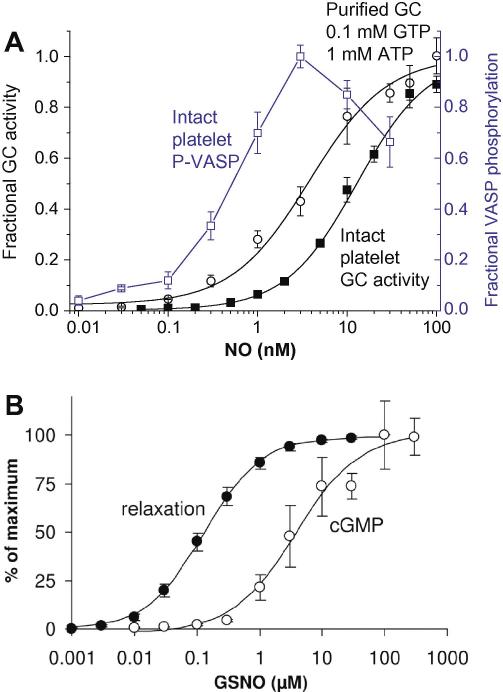
Comparative concentration–response curves for NO or nitrosoglutathione (GSNO) for the stimulation of GC activity and for eliciting downstream effects. (A) Clamped NO concentrations were delivered to intact rat platelets maintained *in vitro* (filled squares) or to purified bovine lung GC incubated with presumed physiological concentrations of GTP and ATP (circles); the resulting GC activity is expressed as a fraction of maximum (left-hand ordinate). The concentration–response curve for cGMP-dependent phosphorylation of vasodilator-activated phosphoprotein (VASP) after exposing rat platelets to NO for 1 min is also expressed as a fraction of the maximum level achieved (open squares and right-hand ordinate). Data are from Roy et al. [47] and Mo et al. [48]. (B) Comparative potency of S-nitrosoglutathione for elevating cGMP (1 min exposure) and eliciting relaxation of rat aorta *in vitro*; from Mullershausen et al. [54] with permission.

A detailed enzyme-linked receptor model for NO-activated GC that accounts for all its main functional properties reported over the years has recently been published [47]. Under the usual biochemical assay conditions, purified GC protein is activated 50% by 1 nM NO. This value depends on the concentration of substrate (GTP) and is also affected by ATP so that, under the presumed physiological intracellular conditions of 100 μM GTP and 1 mM ATP, the EC_50_ increases to about 4 nM (Fig. 2A), a value not far below the 10 nM EC_50_ measured in intact platelets and other cells [48,49]. Despite the high affinity of the receptors for NO, they exhibit surprisingly dynamic behaviour, allowing switch-on and switch-off of cGMP synthesis in a sub-second time scale [50], determined largely by the kinetics of NO binding and unbinding [47,51]. The mid-value for activation by steady NO concentrations (10 nM) is the obvious starting point for considering the likely range of NO concentrations encountered by target cells using this transduction pathway.

In other signalling systems, receptors are often present in excess of those required to generate a maximum response. In the aorta, for instance, only 6% of the available α-adrenoreceptors need to be occupied by noradrenaline to obtain half-maximal contraction [52]. A similar situation appears to apply to NO receptors because, using knockouts of each isoform, either exogenous NO (using S-nitrosoglutathione) or endogenous NO still relaxed aortic smooth muscle cGMP-dependently despite a loss of 94% of GC activity, although the NO donor became less potent than normal [53]. This result implies that GC protein is normally present in large excess of that needed to relax aortic smooth muscle maximally, an arrangement that allows NO concentrations in the lower half of the concentration–response curve for GC activation to be maximally effective. In agreement with this scenario, as illustrated in Fig. 2B, the concentration–response curve for S-nitrosoglutathione-evoked relaxations in normal rat aortae was shifted about 30-fold to the left of the curve describing the rise of cGMP measured after about the same time interval [54]. Similarly, in rat platelets, the NO EC_50_ for stimulation of cGMP-dependent phosphorylation of vasodilator-stimulated phosphoprotein was about 20-fold lower than for stimulation of GC activity in the same cells [48], the maximum effect being seen at 3 nM (Fig. 2A). There have also been attempts to measure the potency of NO for smooth muscle relaxation directly, yielding EC_50_ values of 10 nM [55,56] and 5 nM [57,58], although there may have been uncontrolled losses of NO in these experiments [56]. Using sodium nitroprusside, which maximally releases one NO per molecule, the EC_50_ for relaxing rat aorta was reported as 6.5 nM, reducing to about 0.8 nM when NO synthesis was inhibited [59]. For reducing coronary vascular resistance in perfused rat hearts *in vitro*, the NO EC_50_ was 1 nM [60]. This is probably the best indication of the active NO concentrations needed to increase blood flow since small vascular relaxations from low NO concentrations produce a disproportionately large increase in flow (flow is proportional to the fourth power of the radius of the vessel). Collectively, the evidence indicates that the NO concentrations eliciting physiological responses in vascular smooth muscle and platelets are likely to fall in the range 100 pM–5 nM.

### Tissue cGMP as an index

Attempts have also been made to use tissue cGMP to bioassay the prevailing NO concentration under resting conditions and after imposition of some fairly extreme stimuli. In brain, an established pathway for activation of nNOS is stimulation of NMDA receptors [45]. Maximal stimulation of these receptors in *in vitro* brain slice preparations of cerebellum, however, yielded a level of cGMP that converted to an average NO concentration of only 2–4 nM NO, based on calibrations made using clamped NO concentrations on dispersed cell preparations of the same tissue [61,62]. Applying a similar protocol to slices of striatum following transient simulated ischaemia gave a peak apparent NO concentration of about 1 nM [63]. Even with active iNOS expressed in optic nerve or in hippocampal slice cultures, the evidence suggested that the global NO concentration did not rise to more than 1–2 nM [64,65]. Only when the iNOS-expressing cell population (microglia) in the slice cultures was expanded further (it was already twice the density found *in vivo*) did the prevailing NO become saturating for cGMP generation, signifying a concentration of 10 nM or more [64]. The underlying assumption in this approach is that the tissue NO concentration is fairly uniform, as would be expected theoretically [66] and as was indicated by cGMP immunocytochemistry under similar experimental conditions in the cerebellar cortex [67] and optic nerve [65]. While it cannot be excluded that there existed subregions with higher-than-average NO concentrations, the results suggest that even under potentially pathological conditions, the global NO concentration in brain tissue remains very low, even *in vitro* when scavenging of NO by circulating red blood cells is lacking.

The functioning of axons in the optic nerve *in vitro* is tonically influenced by NO coming from eNOS in the capillary network and acting to stimulate axonal GC activity [68]. Again, based on cGMP immunocytochemistry, this source did not just affect the axons close to endothelial cells but had a pervasive influence throughout the nerve, in accordance with predictions for such a geometric arrangement of sources [69]. Based on the associated level of cGMP relative to the maximum obtainable (2.5%) and assuming an EC_50_ of 10 nM for cGMP generation by NO, the tonic eNOS-derived NO amounts to about 250 pM. A similar number (100 pM) was arrived at for the tonic NO concentration from eNOS that sustained basal cGMP levels and promoted synaptic plasticity in hippocampal slices [70]. These results provide further evidence that cells can transduce endogenous NO concentrations in the subnanomolar range into meaningful biological responses. In the case of the optic nerve, the biological response was a persistent depolarization of the axons apparently mediated through cyclic nucleotide-sensitive ion channels [68].

If NO concentrations in the 100 pM range are biologically active, what concentrations are biologically inactive? The detection limit for transduction of NO signals through the cGMP pathway in cells will depend not only on the amount of GC protein but also on the prevailing phosphodiesterase activity. Estimates of these parameters are available for cerebellar astrocytes and rat platelets [49], from which it can be calculated that NO concentrations of 10 and 30 pM, respectively, could theoretically generate sufficient cGMP (200 nM) to engage targets such as cGMP-dependent protein kinases, so an inactive concentration could be as low as 1 pM.

### Cytochrome *c* oxidase as an indicator

Cytochrome *c* oxidase is inhibited by NO and, as this enzyme is rate-limiting during oxidative phosphorylation, the degree of inhibition of O_2_ consumption that occurs in response to NO has been used to characterise the kinetics of inhibition. Experiments have demonstrated that respiration of various isolated cell types can be inhibited by both exogenously and endogenously generated NO [71–74]. This approach does not, in itself, inform on the physiological NO concentration found in tissues, as dispersed cells have necessarily lost the physiological ratio of NO-producing (e.g., endothelial cells [74]) and NO-consuming cells (e.g., red blood cells, brain cells [75,76]). Theoretically, however, the degree of inhibition of cytochrome *c* oxidase by NO could be used to estimate the concentration of NO in tissues.

NO inhibition of cytochrome *c* oxidase has both competitive and uncompetitive components with respect to O_2_, and is further complicated by the oxidation of NO to nitrite by the enzyme [77]. Several studies have modelled these kinetics in detail [78–80], but it seems that a simple model of purely competitive inhibition is sufficient to account for NO inhibition of O_2_ consumption at the high electron fluxes that reflect actively respiring tissue [79–81]. Eq. (1) therefore represents the relationship between NO and O_2_ concentrations and the rate of O_2_ consumption ($V_{{\text{O}}_{2}}$; as a fraction of the maximum rate), where $K_{{\text{O}}_{2}}$ is 0.2–0.22 μM [79,80] and *K*_NO_ is 0.2–0.25 nM [79,80].

(1) $$V_{{\text{O}}_{2}}=\frac{{\text{O}}_{2}}{{\text{O}}_{2}+K_{{\text{O}}_{2}}\left(1+\frac{\text{NO}}{K_{\text{NO}}}\right)}$$

**Fig. 3:**
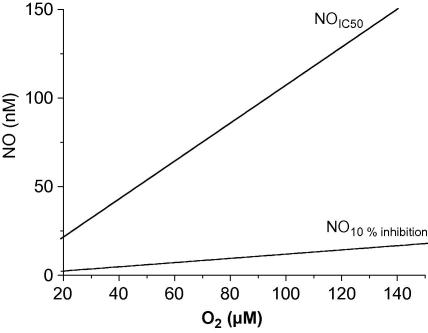
NO concentrations calculated to inhibit cytochrome *c* oxidase by 10% (NO_10% inhibition_) or 50% (${\text{NO}}_{{\text{IC}}_{50}}$) in relation to the O_2_ concentration.

Eq. (1) can be used to calculate both the NO concentration that gives half-maximal inhibition (${\text{NO}}_{{\text{IC}}_{50}}$; Eq. (2)) within the physiological O_2_ range (20–150 μM [82,83]; Fig. 3) and that when O_2_ consumption that is 10% inhibited (NO_10% inhibition_; Eq. (3)).

(2) $${\text{NO}}_{{\text{IC}}_{50}}=\frac{K_{\text{NO}}({\text{O}}_{2}+K_{{\text{O}}_{2}})}{K_{{\text{O}}_{2}}}$$

(3) $${\text{NO}}_{10\text{\%inhibition}}=\frac{K_{\text{NO}}({\text{O}}_{2}+K_{{\text{O}}_{2}})}{9K_{{\text{O}}_{2}}}$$

From these equations, using a mid-range value for $K_{{\text{O}}_{2}}$ (0.21 μM) and *K*_NO_ (0.225 nM), 2–16 nM NO would inhibit respiration by 10%, while 22–150 nM NO would give half-maximal inhibition when O_2_ is in the physiological range (Fig. 3). The NO concentration producing any other change in O_2_ consumption at a given concentration of O_2_ could be also calculated from Eq. (1).

Experimental data are inconclusive as to the degree of NO-mediated inhibition of respiration that occurs physiologically. In brain slices, in which the O_2_ concentration was 0–120 μM, there was no effect of eNOS or nNOS inhibition on basal O_2_ consumption or on O_2_ consumption following stimulation of these NO synthases, and the NO concentration was estimated to be below 6–22 nM [84]. This result is consistent with *in vivo* data, in which NOS inhibition had no effect on cerebral O_2_ consumption or on the redox state of cytochrome *c* oxidase [85–87], and with estimates based on the prevailing level of cGMP in the brain slices (see above). Results in muscle and kidney have been more varied than those in brain. *In vivo*, some studies find no change in O_2_ consumption after NOS inhibition (myocardium and skeletal muscle [83,88,89]), while others report substantial increases (45–55% in skeletal muscle [90,91], indicating that NO inhibits respiration by 30–35%).^1^ *In vitro*, bradykinin-stimulated NO production inhibited O_2_ consumption by 40% in skeletal muscle slices exposed to 150–240 μM O_2_ [91] and by 30% in myocardial segments exposed to 140–220 μM O_2_ [92]. Similarly, O_2_ consumption by kidney *in vivo* was increased by 90% after NOS inhibition (indicating that NO had been inhibiting respiration by 53%), while *in vitro*, NO production from application of bradykinin led to a 10–15% decrease in O_2_ consumption in kidney slices exposed to 200 μM O_2_ [93]. If competitive inhibition of cytochrome *c* oxidase by NO were wholly responsible for these effects, from Eq. (1), the minimum NO concentration *in vivo* would be 10–25 nM (using an estimated tissue capillary O_2_ concentration of 20 μM [94]). *In vitro,* the minimum required NO concentrations would be 100, 60 and 23 nM in skeletal muscle, myocardium and kidney, respectively, if calculated using the lower value of the applied O_2_ concentration in each case. Average tissue slice O_2_, however, must be lower than in the bathing solution because the tissue consumes O_2_, so NO concentrations lower than the values given above would be needed to account for the observed effects. While a direct action of NO on cytochrome *c* oxidase is the simplest explanation for the observations, NO can also decrease respiration via production of cGMP [91,92], making it difficult to extrapolate the physiological NO concentration from the degree of respiratory inhibition alone. The lack of inhibition of respiration in brain, however, sets an upper limit of 22 nM [84]. Most of the experiments on other tissues, furthermore, would only allow an estimate of the average NO concentration across the tissue, unless more specific information was available about the O_2_ and $V_{{\text{O}}_{2}}$ at different depths within it. To get estimates of local NO levels, direct detection of NO would be required or the simple competitive model used here would have to be extended to incorporate the diffusional spread of NO and O_2_ [84].

## Theoretical analyses

Mathematical models have often been used to try to predict the NO profiles generated in the vasculature, brain, lung and other tissues. These studies simulate the diffusion and reaction of NO in biologically relevant geometries, for example by representing an arteriole and surrounding layers of endothelium, smooth muscle and parenchymal tissue as a series of concentric cylinders. Parameters for the rate of production, diffusion and consumption of NO in different cell types are then estimated from experimental data, allowing the spread of NO from its source (e.g., endothelial cells) to its targets (e.g., smooth muscle cells) to be calculated.

Many of these studies have generated interesting predictions as to the spatial and temporal nature of endogenously produced NO signals. For example, Philippides et al. [95] demonstrated that a plexus of very fine nerve fibres, such as the NOS-expressing processes found in mammalian cerebral cortex, are optimal for generating a volume NO signal that is tightly linked in space and time to the region of neuronal activity, a situation of possible relevance to NO-dependent learning [96]. Models of arteriolar NO production have been informative, for example, in illustrating the critical reduction in smooth muscle NO levels when free haemoglobin escapes from erythrocytes, providing a graphic explanation for the hypertension seen after haemolytic diseases or after use of haemoglobin-based oxygen carriers [97,98]. Some models have also incorporated O_2_ diffusion and reaction, considering the O_2_ dependence of NO synthesis and some forms of NO consumption, and the potential inhibition of O_2_ consumption by NO [99,100]. Other models, however, suggest that while NO levels are sensitive to the amount of O_2_, they do not reach high enough concentrations to affect O_2_ consumption [84].

There is a great deal of variation in the levels of endogenous NO predicted by these models, ranging from 60 pM to 1 μM in a single active neuron [66,76], and from 0.1 to ∼300 nM in arteriolar smooth muscle [101,102]. The reason for these wide disparities is largely due to the choice of values for one critical parameter: the NO synthesis rate. In the absence of direct information, the majority of models [66,69,95,100,103–107] chose an NO synthesis rate that achieved NO concentrations reported by Malinski, namely 1 μM NO on the surface of a single stimulated endothelial cell [31] and a gradient of 0.45 μM NO between the endothelial and smooth muscle layers of rabbit aorta [108]. Correspondingly, these studies estimate that endogenous NO concentrations are in the same range, decreasing to 100–300 nM in the presence of NO consumption by blood. While these concentrations agreed with an early tentative estimate of the potency of NO for activation of GC, (EC_50_ < 250 nM; [109]), as discussed above, experiments using stable concentrations of NO have demonstrated that the sensitivity range of for cellular cGMP production and vessel relaxation is actually much lower (EC_50_ ∼ 10 nM; [47,56]). The large discrepancy between prediction and experimental evidence on the NO concentrations that are functionally active has caused some consternation as to how vessels can avoid being maximally dilated in basal conditions [110]. As discussed above, however, the porphyrinic sensor developed by Malinski reacts with a number of biological species and appears to overestimate endogenous NO concentrations by 2–3 orders of magnitude.

Other models have used measurements of NOS activity, rather than the NO concentration, to calculate the endogenous NO production rate. These models predict much lower endogenous levels of NO. Hall and Garthwaite [76] used the rate of NOS activity measured in homogenised cerebellum [111] and predicted endogenous NO concentrations of <10 nM if NO production was activated throughout the tissue, or 10–30 pM if a single neurone was activated. Chen and Popel [101,112] used the kinetic models of NO production from eNOS and nNOS formulated by the Stuehr group [113,114] together with estimates of the cellular concentrations of the two NOS proteins. They predicted that NO levels in vascular smooth muscle would be up to 100 pM if eNOS alone were active and 4 nM at physiological O_2_ levels (100 μM) if perivascular nerves were also synthesizing NO.

**Fig. 4:**
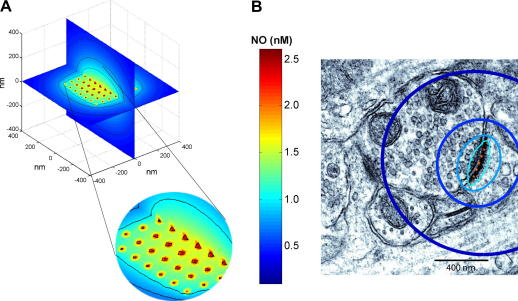
Predicted steady-state NO concentration profile at a synapse. (A) The NO sources are assumed to be a 7 × 7 array of NOS molecules, each producing 20 NO molecules/s (based on the initial rates of NO formation reported by Santolini et al. [114] after correcting for temperature). Each NOS molecule is assumed to be 100 Å (10 nm) in diameter [171]. Contour lines show NO concentrations of 0.5, 0.75, 1, and 2 nM NO. (B) Contour lines shown in (A) for the position perpendicular to the plane of the synapse (with an additional line at 0.25 nM NO) are superimposed on an electron micrograph of a typical excitatory synapse (from [115], reprinted with permission from AAAS; panel modified from [45]).

For central synapses, an alternative approach is depicted in Fig. 4. nNOS in synapses is frequently physically associated with NMDA receptors and is activated when NMDA receptor channels open, allowing an influx of Ca^2+^. Each NMDA receptor can only associate with one nNOS molecule and a typical postsynaptic membrane may contain up to 50 NMDA receptors [115] and, therefore, may have associated with it a similar number of nNOS molecules. Taking the nNOS molecules to exist in a 7 × 7 array and the extreme case of all of them being simultaneously active, and generating NO at the highest rate achievable from measurements of the activity of purified nNOS after adjusting for temperature (20 s^−1^, [114]), the steady-state profile predicts an NO concentration of only 1 nM just the other side of the synapse (60 nm away from the central source where the NO concentration is highest), falling to 250 pM within a distance of 1 μm (Fig. 4B). Even within the source itself (assumed to be a sphere of diameter = 10 nm), the NO concentration reaches only 2.5 nM. Unless the activity of nNOS is radically underestimated, it is difficult to understand how NO concentrations higher than these could exist physiologically during normal synaptic transmission or when NMDA receptors become activated more persistently, as occurs during prolonged high-frequency synaptic stimulation. Indeed, as reported by Salerno [116], following persistent activation, nNOS is predicted to generate NO in a biphasic manner, with a peak activity after ∼50 ms followed by a 3- to 4-fold decrease to a steady-state level, further emphasizing that the NO concentration profiles shown in Fig. 4 are likely to be upper limits.

Because the estimates of NO production used in this and in the analyses of Chen and Popel [101,112] do not derive directly from physiological conditions, they are unlikely to be completely accurate, but they have the advantage of making no prior assumptions about the NO concentration that ought to be produced. The resulting predictions concerning endogenous NO concentrations are within range of the NO concentrations measured with some electrode designs and fall within the sensitivity range for GC activation.

## NO concentrations *in vivo* versus *in vitro*

Many experiments are carried out *in vitro*, on cultured or dispersed cells or tissue slices on the assumption that the properties of NO signals reasonably reflect the *in vivo* situation. The size and shape of NO signals are determined by the balance between the rates of NO synthesis, consumption and diffusion, but *in vitro* experiments are often performed under conditions in which the rates of synthesis and consumption are substantially different from those existing *in vivo*, limiting the degree to which we can infer physiological NO levels from those measured *in vitro*. Even the NO diffusion coefficient, which most studies have taken as the value in aqueous solution, 3300 μm^2^/s [108], has been measured to be 4-fold lower in tissues [117], leading to a reduced diffusional spread of NO from its source and, hence, a higher local NO concentration.

### NO production

The rate of NO synthesis, as discussed above, is related to the O_2_ concentration, which ranges from ∼20 μM in brain [82] to ∼150 μM in arterial blood [83]. eNOS and nNOS have very different O_2_-dependencies (apparent *K_m_* values = 4 μM and 350 μM, respectively [8]), so the rate of NO production from nNOS is sensitive to the O_2_ concentration within the physiological range, while eNOS is not. iNOS has an intermediate O_2_ dependence (apparent *K_m_* = 130 μM [8]). Importantly, however, *in vitro* experiments are usually conducted using solutions at air-equilibrated O_2_ levels (210 μM O_2_), or even gassed with 95–100% O_2_ (1 mM O_2_). In cell monolayers at 210 μM O_2_, the rate of NO production from nNOS and iNOS will be about 10-fold and 5-fold higher, respectively, than at a physiological O_2_ concentration within parenchymal cells of, say, 20 μM. In tissue slices, the situation is more complex, as O_2_ consumption by the tissue generates an O_2_ gradient across the slice, such that the edge of the slice experiences supraphysiological O_2_ levels, while the centre may be normoxic or even hypoxic [84]. This means that the rate of NO production from nNOS and iNOS will differ across the thickness of the tissue slice, the average rate differing with the bathing O_2_ concentration and the rate of O_2_ consumption by the tissue. As physiological O_2_ levels are well above the apparent *K_m_* of eNOS for O_2_, in *vitro* rates of NO production from eNOS should better reflect the *in vivo* production rates.

### Enzymatic NO consumption

Several pathways have been proposed to participate in consumption of NO. The reaction of NO with oxyhaemoglobin to form methaemoglobin and nitrate is very fast and, although this reaction is slowed in blood by the encapsulation of haemoglobin in erythrocytes, the rate constant for NO in blood is 350–6500 s^−1^ [75,102], yielding a luminal half-life of 0.1–2 ms. *In vitro* preparations do not have circulating red blood cells and the absence of this powerful sink for NO will increase the size and duration of *in vitro* NO concentration profiles compared to *in vivo*, particularly, of course, in blood vessels themselves where most of the NO synthesised is inactivated by erythrocyte haemoglobin [103]. For parenchymal cells, the main blood supply is in the capillaries, which typically represent about 2% of the tissue volume [118]. For NO derived from capillary endothelial cells, reaction with erythrocyte haemoglobin has been calculated to give rise to an inactivation rate constant of 10–20 s^−1^ [69]. For NO generated in the parenchyma, the corresponding rate is calculated to be about 1 s^−1^ when the geometry of the capillary circulation is taken into consideration and assuming that any NO entering a capillary is lost (Wood and Garthwaite, unpublished result).

The extent to which the lack of inactivation by circulating erythrocytes in *in vitro* preparations will affect the NO concentration will depend on the activity of other NO consumption pathways. Tissue slices of a brain region (cerebellum) consumed NO with an apparent maximal rate of 0.2–2 μM/s and a *K_m_* of 10 nM [76,84], meaning that 10 nM NO would decay with a rate constant of 12–120 s^−1^, equivalent to a half-life of 6–60 ms. The NO inactivation rate in optic nerve appeared to be similarly high [65]. Hepatocytes consumed NO *via* a process that depended linearly on both O_2_ and NO, such that the parenchymal half-life of NO would be 0.09–2 s, depending on the O_2_ concentration [99]. The identities of the enzymes responsible for these processes are not yet clear, but cytochrome P450 oxidoreductase is implicated in at least a component of NO consumption by brain and a cancer cell line [119,120], and a similar mechanism seems to be present in endothelial cells [121]. Other enzymes reported to consume NO include lipoxygenases and prostaglandin H synthase [122,123], cytochrome *c* oxidase [124], myoglobin [125], and cytoglobin [126]. Further characterisation of the processes that are most relevant for physiological NO inactivation in different tissues is required to assess how NO consumption will differ *in vitro* and *in vivo*, but the O_2_ dependence of the hepatocyte pathway would suggest that NO consumption is faster in the hyperoxic conditions commonly used *in vitro*. Where nNOS is the source of NO, this increase in NO consumption would not greatly affect the resulting NO concentration, as the rate of NO production would also be increased, whereas if NO was generated from eNOS, which is not O_2_ dependent above ∼20 μM, the NO concentration achieved would fall.

Not all cells are equipped with a mechanism for rapidly consuming NO. It may be no coincidence that cells of the cardiovascular system other than erythrocytes appear deficient. This applies to platelets and mixed white cells isolated from rat blood [127] as well as to intact rat aorta [117], although cultured endothelial cells were found to inactivate NO [121]. Hence, measurements of the potency of exogenous NO for relaxing aorta (EC_50_ = 1–10 nM; see above) may be underestimated but not to the extent that occurs in other tissues, such as liver or brain. Here, NO consumption gives rise to substantial NO concentration gradients across the tissue when it is applied in the bathing medium [76], such that high NO concentrations must be used in order to achieve low levels of NO at the centre of the tissue block. In intact brain slices [76] and optic nerve [65], for example, the apparent EC_50_ of NO for cGMP accumulation at steady-state is about 1000-fold higher than when NO has direct access to its target cells in isolation. Thus, the EC_50_ for NO measured in such tissues reflects the properties of the inactivation mechanism rather than the sensitivity of the constituent cells to NO, much as when glutamate and other transmitters that are subject to avid removal from the extracellular space are administered in the bathing medium [128]. One disadvantageous experimental consequence is that when sufficient NO is given to stimulate GC in the centre of a block of tissue, the applied NO may be vastly supraphysiological at the edges of the tissue, where it could have pathological effects, such as respiratory inhibition. In brain slices, for example, when 1 μM NO was applied to 400 μm thick brain slices bathed in 1 mM O_2_, only half of the maximum possible cGMP was generated [76]. This result indicates that NO was submaximal for stimulation of GC at the centre of the slice (<20 nM) but, at its surface, NO would inhibit mitochondrial respiration by 50%.

Conversely, if cell monolayers are used as the experimental material, a loss of NO inactivation could lead artifactually to the accumulation of toxic levels of NO. For example, if NO were generated continuously in a three-dimensional tissue at a global rate of 10 μM/min, the resulting steady-state NO concentration would be 1–2 nM if NO were consumed at the rate estimated for intact brain tissue [76]. With the same cells dispersed in monolayers at a dilution of 1:100 (i.e., at approximately 1 mg protein/ml, now generating 100 nM NO/min) with NO being consumed only through autoxidation (in an air-equilibrated medium), the resulting NO concentration would be near 1 μM, which is expected to inhibit respiration by more than 80%.

### Chemical NO consumption

In addition to enzymatic NO metabolism, NO can react rapidly with other radicals such as superoxide (O_2_^•^^−^), thiyl radicals (RS^•^) and lipid peroxyls (LOO^•^) [129–131]. The concentrations of these radicals are unlikely to be high enough normally *in vivo* to contribute significantly to NO consumption in physiological conditions. Superoxide is normally efficiently scavenged by superoxide dismutase [132], but its reaction with NO is more likely to occur when it is produced in larger amounts, for example on reperfusion after ischaemia or during inflammation [133]. Lipid peroxyls are increased in aged animals and in diseases such as atherosclerosis and neurodegeneration [134]. In addition, their formation increases on tissue disruption [134] and so they can artifactually generate a sink for NO *in vitro* [127]. Thiyl radicals, which react with NO to produce nitrosothiols (RSNO) [135], are produced during oxidative stress and after reaction of DNA, peroxyl and tyrosyl radicals with glutathione (GSH) [136]. In a macrophage cell line expressing iNOS, however, only a small proportion of endogenously produced NO ended up as nitrosothiol (0.02% [137]), indicating that this reaction is unlikely to contribute much to NO consumption. It could, however, be artificially exacerbated *in vitro*, as Fe^3+^ and Cu^2+^, which are common contaminants of experimental buffer and salt solutions [138], cause increased thiyl radical formation [136].

## Concluding remarks

Leaving aside the incongruous findings made using many electrode designs, theory and experiment are converging to indicate that NO operates physiologically at concentrations that are orders of magnitude lower than the near-micromolar once considered correct. From the evidence, a range of 100 pM–5 nM appears a reasonable one to settle on although, in principle, NO down in the low pM range could elicit biological responses in cells through GC activation. If the range is correct, much of the chemical and biological reactivity of NO seen at higher NO concentrations becomes of doubtful physiological relevance. Moving towards pathology, on examination of conditions suspected to show a build-up of higher NO concentrations, including inflammation and excitotoxic neurodegeneration, no such build-up was detected in intact brain tissue. That higher concentrations can be found under similar conditions in dispersed cell models may be attributed to an artificial loss of NO consumption in such preparations. Nevertheless, two important caveats should be raised. One is that the results of some studies (but not others) on some peripheral organs (but not brain) are consistent with NO existing *in vivo* at concentrations capable of inhibiting mitochondrial respiration significantly. Whether the inhibition is a purely direct effect of NO (necessitating NO concentrations in excess of 5 nM) or includes an indirect, possibly cGMP-mediated, component needs clarification. The other uncertainty concerns the NO concentrations existing within a concentrate of NO-generating cells, for example macrophages or microglia expressing iNOS in inflammatory foci. As indicated by *in vitro* results, NO could rise to greater than 10 nM in such a scenario although whether it does or not *in vivo* where the O_2_ concentration is lower (reducing the rate of NO synthesis) and where there is tissue perfusion (allowing NO consumption by the blood), remains to be studied, as does the relevance of such NO concentrations to humans, in which iNOS-expressing cells appear to produce much less NO than their rodent counterparts [139,140]. The establishment of trustworthy methods for direct microelectrode measurement of NO concentrations, and the (more foreseeable) progression of the newly developed NO biosensors for quantitative imaging of NO signalling in subcellular dimensions and in real time to tissues *in vivo*, will facilitate advance in this fundamental, but still unsettled, area.

## Figures and Tables

**Table 1:** Electrode measurements of NO concentrations in different tissues.

Preparation	*In vivo/vitro*	Stimulation	[NO] (nM)	Reference
*Vasculature*				
Dog femoral artery	*In vitro*	Acetylcholine	2.5	[11]
Rat mesenteric artery	*In vitro*	Acetylcholine	20	[141]
Rabbit coronary artery	*In vitro*	Acetylcholine	295	[142]
Rat aorta	*In vitro*	Acetylcholine	390	[12]
Human radial and mammary artery	*In vitro*	Acetylcholine	15 (both)	[143]
Human radial artery	*In vitro*	Bradykinin	6	[143]
Human mammary artery	*In vitro*	Bradykinin	20	[143]
Porcine pulmonary artery	*In vitro*	Bradykinin	160	[144]
Porcine pulmonary vein	*In vitro*	Bradykinin	103	[144]
Porcine aorta	*In vitro*	Bradykinin	450	[31]
Porcine aorta	*In vitro*	Bradykinin + indomethacin	105	[145]
Rabbit aorta	*In vitro*	Carbachol	17	[146]
Rabbit femoral artery	*In vivo*	Ischaemia	140	[147]
Rat mesenteric artery	*In vitro*	Noradrenaline	5	[148]
Rat aorta	*In vitro*	Substance P	63	[149]

*Brain*				
Rat medial prefrontal cortex	*In vivo*	Cocaine	8	[150]
Rat somatosensory cortex	*In vivo*	Electrical stimulation	126–190	[151]
Rat auditory cortex	*In vitro*	Electrical stimulation	0.38	[152]
Rat cerebellum	*In vitro*	Electrical stimulation	2.2	[153]
Rat cerebellum	*In vitro*	Electrical stimulation	4	[154]
Rat striatum	*In vivo*	Electrical stimulation	2.5	[155]
Rat striatum	*In vivo*	Electrical stimulation	3	[156]
Rat striatum	*In vivo*	Electrical stimulation	4.5	[28]
Rat striatum	*In vivo*	Electrical stimulation	8	[27]
Guinea pig laterodorsal tegmental nucleus	*In vitro*	Electrical stimulation	33	[157]
Mouse olfactory bulb	*In vitro*	Electrical stimulation	100	[158]
	*In vivo*	Odorant	18–84	
Rat cortex	*In vivo*	Impact injury	83	[159]
Rat cortex	*In vivo*	Ischaemia	1000	[14]
Rat cortex	*In vivo*	Ischaemia	17	[13]
Cat nucleus tractus solitarius	*In vivo*	l-Arginine	3	[30]
Rat cerebellum	*In vitro*	NMDA	1800	[160]
Cat ventrolateral medulla	*In vivo*	NMDA	1.1	[29]
Rat cerebral cortex	*In vivo*	NMDA	20	[161]
Rat striatum	*In vivo*	NMDA	176	[162]
Rat striatum	*In vivo*	NMDA	520	[149]
Rat hippocampus	*In vitro*	NMDA	250	[20]
		Glutamate	350	
Rat hippocampus	*In vivo*	NMDA	400	[163]

*Heart*				
Rabbit heart	*In vitro*	Basal	1200	[164]
Rat heart	*In vitro*	A23187 (Ca^2+^ ionophore)	650	[147]
Dog coronary sinus	*In vivo*	Acetylcholine	7	[165]
Guinea pig heart	*In vivo*	Bradykinin	21	[166]

*Other*				
Mouse kidney	*In vitro*	Basal	70–200	[167]
Rat kidney	*In vitro*	Basal	0.15	[168]
Rat Lung	*In vivo*	LPS	140	[147]
Guinea pig trachea	*In vitro*	Bradykinin	0.002	[9]
Guinea pig bronchiole	*In vitro*	Bradykinin	0.0004	[9]
Guinea pig myenteric ganglion	*In vitro*	Nicotine	46	[169]
Guinea pig circular muscle	*In vitro*	Nicotine	124	[169]
Rat spinotrapezius muscle	*In vitro*	Acetylcholine	300	[170]

## Footnotes

1 An increase in $V_{{\text{O}}_{2}}$ on removal of NO of 45% means that the new, uninhibited respiration rate is 145% of the rate when inhibited by NO. The respiration rate in the presence of NO is therefore (100/145)*100% = 69% of the uninhibited rate, i.e., a 31% inhibition.
